# Sociodemographic relationships of motivations, satisfaction, and loyalty in religious tourism: A study of the pilgrimage to the city Mecca

**DOI:** 10.1371/journal.pone.0283720

**Published:** 2023-03-30

**Authors:** Tahani Hassan, Mauricio Carvache-Franco, Orly Carvache-Franco, Wilmer Carvache-Franco

**Affiliations:** 1 Brunel Business School, Brunel University London, Uxbridge, London, United Kingdom; 2 Universidad Espíritu Santo, Samborondón, Ecuador; 3 Facultad de Economía y Empresa, Universidad Católica de Santiago de Guayaquil, Guayaquil, Ecuador; 4 Escuela Superior Politécnica del Litoral, ESPOL, Facultad de Ciencias Sociales y Humanísticas, Guayaquil, Ecuador; University of Naples Federico II: Universita degli Studi di Napoli Federico II, ITALY

## Abstract

Religious tourism is a growing sector of the tourism market because of the many social and cultural changes in the 21st century. Pilgrimage centers worldwide are considered important at the levels of religion, heritage, and culture of tourism. Despite the popularity of journeys to pilgrimage centers and their global importance, there is still a lack of knowledge about the dimensionality and impact of socio-demographic factors on visiting these centers. This study aims to (i) establish the motivational dimensions of the pilgrimage to Mecca (ii) identify the relationship between socio-demographic aspects of pilgrims and the motivation (iii) determine the relationship between socio-demographic aspects of pilgrims, satisfaction, and loyalty. The research was carried out on pilgrims who had visited Mecca. The sample consisted of 384 online surveys. Factor analysis and multiple regression method were applied to a analyze data. The results show three motivational dimensions: religious, social, and cultural, and shopping. Additionally, there is evidence of a relationship between age, marital status and average daily expenditure per person with some motivational variables. Similarly, a relationship was found between average daily expenditure per person and other variables such as satisfaction and loyalty. This study helps tourism companies pay attention to pilgrims’ the socio-demographic characteristics of and match them with their motivation, satisfaction, and loyalty during the planning process.

## 1. Introduction

Religious tourism is a growing form of travel due to the many socio-cultural changes taken place, including globalization, transport development, and commercialization [1]. Religious tourism is the journey to sacred sites to meet the religious and spiritual needs of the tourists traveling to those destinations [2–5].

The most common form of religious tourism is a pilgrimage which involves practices of the religion such as Hajj to Mecca in Islam, pilgrimage to the ministry of Jesus and sites associated with saints for Christians, pilgrimage of Jews to the City of Jerusalem, and pilgrimage of Buddhists to temples and the Ganges River which occur at specific times of the year [3].

Traveling to religious destination is influenced by tourist’s motivations. Those reasons are why tourists to visit the site [6]. Generally, motivations are defined as the reasons behind visiting a specific destination which has a relevant effect on the design, promotion and planning of travel destination [7, 8]. Religious tourism is generally divided into religious motives such as worship and secular motives, including cultural motives, relaxation motives, pleasure-seeking motives such as shopping, seeing old historical sites, having peace, and spending times with family and friends [9]. Previous studies found that religious tourists’ motivation can be influenced by three other factors: socio-demographic characteristics, satisfaction, and loyalty. First, tourism socio-demographic aspects of are tourists’ properties or attributes of such as gender, age, educational level, job, income, region, and nationality [3, 10]. It is crucial to study those aspects because of their role in explaining the differences in tourists’ lifestyles and travel motivations hand and their impact on the tourists’ demand and tourism services [10–12]. The socio-demographic characteristics of tourists were also found to have a significant impact on families’ travel plan, behaviour, and expenditures [13]. Second, satisfaction addresses how the destination matches the needs of tourists [14]. Third, loyalty to a tourist destination refers to the willingness to recommend the destination to other people and make a future revisit [15]. Tourists’ satisfaction and loyalty are essential considerations for tourism marketers in improving the quality and services of tourist interests and ensuring a delightful experience at a destination [14].

The pilgrimage to Mecca is one of the most important religious events in the world due to the gathering of millions of people from all over the world in the Holy city of Mecca in Saudi Arabia during a specific time of the year to perform Hajj [16–18]. However, the pilgrimage to Mecca takes two forms: Hajj and Umrah. The Hajj takes place in the last month of the Islamic calendar, and it is obligatory for every Muslim once in life, whereas the Umrah is performed at any time of the year [19, 20]. During the visit to Mecca, Muslims pray in the Grand Mosque (Masjid al-Haram) and visit religious sites such as Safa and Marwa Mountain in order to pray, find peace, happiness and follow Prophet Muhammed (PBUH) on his last visit to Mecca [21].

A careful look into the previous studies about religious tourism showed a paucity of studies about the relationship of socio-demographic factors with motivation, satisfaction, and loyalty among pilgrims to religious sites. Studying this interrelationship is vital for preparing tourism plans, improving services, accommodating pilgrims, and meeting their needs since they will be satisfied and repeat their visits in the future. Thus, the objectives of this study are to (i) establish the motivational dimensions of the pilgrimage to Mecca (ii) determine the relationship between socio-demographic aspects of pilgrims and their motivation (iii) identify the relationship between socio-demographic aspects of pilgrims, satisfaction, and loyalty.

## 2. Literature review

### 2.1. Motivations in religious tourism

Religious tourism is an old form of tourism that appears and developed with the religions throughout history [22]. Religious tourism is s the visit to a specific destination, and it includes three categories: (1) sites of high value that attract large numbers of tourists such as cathedrals, (2) religious festivals and (3) pilgrimages shrines [23]. Furthermore, Nyaupane et al. [24] ascertained those visitors to religious sites tend to travel with their families or organized groups during specific seasons influenced by climate, holidays, work schedules, and ceremonies. Pilgrimage, the most common form of religious tourism, is a practice in some religions such as Islam, Christianity, Buddhism, and Hinduism [3].

Previous studies about tourists’ motivation agreed on two types: push and pull motives. Push motives are tourists’ internal desires for having fun, relaxation, family bonding, social interaction, knowledge seeking, and enjoying challenges. Pull motives are external factors that attract tourists to travel to a particular destination, including the historical, cultural and environmental qualities and attributes [25–29].

Studies have identified different motives for religious tourism. These motives include the need to search for life meaning and develop personal values [1]. Thus, people travel to religious sites to search for the meaning of life, spend time with family and friends, visit historical and cultural place, and relax [30, 31]. These motives are classified into three main groups: religious, touristic, and recreation [32]. However, previous studies showed that motivations vary according to the type of the religious site. For instance, churches are considered cultural buildings visited for less religious reasons. Hence, churches are visited for historical and cultural motives more than religious ones [33–35]. Concerning Buddhist sites, few studies explored tourists’ motives. Scholars found push motives such as religious belief and mental relaxation and pull motives such as seeing the architecture, cultural enjoyment, and history [36, 37]. Finally, for Muslim pilgrims to Mecca, their motives are not limited to religion. Other motives include praying in the Masjid al-Haram (The Great Mosque of Mecca), leisure, shopping, and benefitting from the Islamic tourist packages [38, 39].

Regarding the nature of the pilgrims and their motivations. For Muslims, Luz [40] stated that there are a variety of reasons why Muslims travel to Mecca and Medina in Saudi Arabia for pilgrimage. The benefit of receiving forgiveness of sins is the primary incentive for praying in Masjid al-Haram (The Great Mosque of Mecca) and Masjid-e-Nabawi in Medina. Finding love and pleasure is the second justification. Because of their devotion to the Prophet Muhammad, who visited these locations, Muslims make pilgrimages to Mecca. In order to express their love for the Prophet, Muslims appreciate and cherish certain locations. Third, Muslims travel there on pilgrimages, which enables them to respect other travellers’ cultural and ethical beliefs as well as their own. These travellers come to perform the Hajj in Mecca and to see Masjid-e-Nabawi in Medina [21].

In Christianity, churches are regarded as cultural sites in Western nations and are frequently visited by tourists [35]. Tourists are more likely to visit churches for historical and cultural reasons than for religious ones, according to several UK surveys [41]. A similar analysis of English visitors to Chichester Cathedral by Gutic et al. [33] revealed that history, architecture, and curiosity were the primary motivators. However, spiritual motivations like prayer or inner serenity were less significant. St. David’s Cathedral in Wales was researched by [42] who agreed that people go to the cathedral to learn more about the national heritage.

Regarding Buddhism there aren’t many studies on Buddhists sacred sits that examine the motivations behind visiting Buddhist locations. For instance, Bodhgaya, an Indian Buddhist site, was researched by Piramanayagam et al. [37] in 2021. Three factors were discovered by this study: religious convictions, history, architecture and culture, and service quality. They claimed that the best reason to travel to Bodhgaya was due to the caliber of the services offered. Religious convictions and mental rest were identified as driving motives in Wang et al. [36]’s study on religious tourism motivation in Buddhist Mountain (China), whereas cultural enjoyment was identified as an attracting factor. The aforementioned discussion confirmed that there is a difference among religions in regard to their motivations to visit religious sites. This could be attributed to the difference of beliefs and the nature of these religions.

### 2.2. Socio-demographic aspects and motivations in religious tourism

Religious tourism is studied from the socio-demographic aspect. However, research on this relationship is scarce, and it needs to be studied to understand these concepts better and improving the services to the pilgrims of those sacred sites. Studies found a difference between men and women in travelling for religious tourism [23]. For instance, visits to saints and the cult of the Virgin Mary are known for recovering from illnesses, disease protection and resolving infertility; thus, these sites are more visited by women than men. For example, many visitors to European Marian shrines are female women pilgrims who make vows for health issues related to them or family members, especially children [12]. Similarly, most pilgrims to Mecca are male because of travel gender limitations between men and women [43]. Variations are found in the relationship between socio-demographic aspects and motivation in religious tourism. For example, Vistad et al. [44] studied pilgrims’ visits to Nidaros Cathedral in Trondheim, Norway, and found no motivational differences among pilgrims based on their level of education whereas the Homeland/ region influences nature knowledge and joy motivations that more important to European pilgrims than to Norwegians. Being in solitude is more important to European and German pilgrims than to Norwegians. The study showed that being in solitude is less important for inexperienced pilgrims. Finally, experienced pilgrims are more religiously motivated than less experienced ones. Hence, pilgrims’ socio-demographic aspects of significantly impact their motivations to travel to sacred destinations. Pillai et al., [45] investigated the pilgrims who visited the 17th Exposition of St Francis Xavier’s holy relics, a Christian religious event in Goa, Indonesia and found no relationship between pilgrims’ motivation and their demographic aspects except age. Another study conducted by Liro [1] about the Roman Catholic sanctuaries in Poland found that demographic aspects have a significant impact on religious tourism. Women are more motivated to travel, pray and participate in cultural events than men. This study also found a difference between the travel motives of Polish, and foreign visitors. Polish and foreign tourists were motivated for praying and pilgrimage whereas foreign visitors of these sacred sites as part of their sightseeing in the region. Finally, in terms of gender, older people are more religiously motivated than young ones.

Studies about the relationship between sociodemographic aspects and motivations of pilgrims are not conclusive and lead to different results. Thus, further investigating the relationship between socio-demographic aspects and motivations among pilgrims could provide knowledge about their travel preferences. This study could also assist the religious tourism market plan future trips to fit pilgrims’ motivations and their socio-demographic characteristics.

Therefore, based on the discussed arguments, the following research question is derived:

*Q1: What is the relationship of socio-demographic aspects with motivation to visit Mecca?*

### 2.3. Socio-demographic aspects related to satisfaction and loyalty in religious tourism

Satisfaction is essential for tourists’ companies to successfully market destinations and improve their quality of services and products. Satisfaction is crucial for improving the destination image [46, 47]. Furthermore, satisfaction can provide forecasts for the loyalty to a particular destination and the desire of the tourists to return to that destination [48]. In general, tourism satisfaction is the positive feeling about the expectations and the perceived benefits that tourists express after travelling [49]. Another crucial element in tourism is loyalty which is essential in improving the travel destination image through positive word of mouth. Loyalty is the repetition of visit to a destination and visitors’ recommendations of the destination [50]. Regarding religious tourism, satisfaction is the degree to which the destination matches the need of the tourist [51]. Loyalty in religious tourism is overall feeling about the visit, the recommendation of a destination to other people and the intention to revisit the destination in the future [51].

Empirical studies of the relationship between socio-demographic aspects and pilgrims’ satisfaction showed different results. For instance, age, gender, occupation and academic status do not significantly affect on satisfaction among Malaysian Hajj (Pilgrims to Mecca) [52]. Conversely, Joseph et al., [53] found that satisfaction varied among pilgrims to Sabarimala hill temple, Kerala State of India. The researchers found that pilgrims from the agriculture sector have a high level of satisfaction. Pilgrims with high and low income are more satisfied than those with average income. Another study about tourists participating in the “Saint Parascheva” pilgrimage, Romania, found that age variable is the only one among socio-demographic aspects that influenced the pilgrims’ satisfaction, which is attributed to the high level of faith among them [54]. Moreover, religious tourism does not indicate a relationship between sociodemographic variables and loyalty. For example, a study about the antecedents of tourist loyalty in Jammu and Kashmir, India, found that gender moderates the effect of destination image on the satisfaction and loyalty of tourists [55]. Hence, we can infer that socio-demographic aspects may relate to satisfaction and loyalty. However, this relationship may vary from one socio-demographic aspect to another.

Therefore, we propose that socio-demographic aspects of pilgrims may affect their satisfaction and loyalty:

*Q2: What is the relationship between socio-demographic aspects of pilgrims and satisfaction?*

*Q3: What is the relationship between socio-demographic aspects of pilgrims and loyalty?*

## 3. Study area

Mecca is located in the West of Saudi Arabia near the city of Jeddah. Muslims are obliged to visit Mecca once in their life to perform Hajj during the period of 8 to 12th Dhu al-Hijjah (the last Islamic month). Muslims also visit Mecca to perform Umrah, a non-compulsory ritual that can be performed any time of the year [56]. Travelling to Mecca stems from the fact that performing Hajj is the peak of the Muslim pilgrims’ religious life, and it is a representation of the Muslim principles of unity and equality since pilgrims to Mecca perform the same rituals and wear the same cloths [40].

Visiting the holy city of Mecca involves several Islamic sites to perform Hajj or Umrah. The most important is the Great Mosque of Mecca (the Masjid al-Haram), where Muslims must circumambulate the Kaaba. Then, Muslims walk between the mountains of Safa and Marwah. Moreover, Muslims can visit other sacred sites, including Maqam Ibrahim (the stone where Abraham stood when building the Kaaba) and Jable Al- Nour, where Prophet Muhammad received the first revelation in Hera Cave [21]. Pilgrims can visit cultural sites like the Mecca Museum and an exhibition of two sacred mosques while on their trip to Mecca. Al-Zahir Palace was originally known as the Mecca Museum. It has an exhibit on Islamic calligraphy, a chamber dedicated to Islamic art, and a collection of pre-Islamic era archeological finds. The ancient golden metal gate of the Ka’bah of Al Masjid Al Haram in Mecca and the Mosque of the Prophet in Medina are both on display in the Two Mosques Exhibition together with marble emblems, pillars, historical images, and other artifacts. The Ka’bah fabric was created in the Al-Kiswah (cloth of the Ka’bah) Factory. Visitors interested in antiques can also go to the Mecca auction. It offers a fantastic collection of jewels and ancient Arabic coins where people can place bids on the objects on exhibit. The Al-Zaher Palace Museum is another option for them. This museum displays artifacts from various Islamic historical periods in the region as well as the history of Mecca. It was built in an Islamic style in 1944. It was initially used by King Abdul Aziz as his Mecca headquarters, where he met representatives of Muslim pilgrims from all around the world. Then, it was changed into a museum dedicated to Islamic history. Pilgrims can visit the many eateries in Mecca for entertainment and shopping. Paradise Restaurant, which is near to Al Masjid Al Haram, is one of the eateries. It serves conventional cuisines and is open at night. Al Tazaj is another well-known eatery that is well-known throughout the kingdom of Saudi Arabia. It is renowned for serving a variety of BBQ dishes, including hamburgers. Tourists can visit Mecca Mall for shopping. It is a sizable shopping mall that provides families with a calm and welcoming atmosphere. It provides both domestic and foreign product brands, supermarkets, and eateries [57].

## 4. Methodology

This study has the objectives (i) to establish the motivational dimensions of the pilgrimage to Mecca, (ii) to identify the relationship between socio-demographic aspects of pilgrims and motivation (iii) to determine the relationship between socio-demographic aspects of pilgrims, satisfaction, and loyalty. Motivation, satisfaction, and loyalty were the dependent variables and sociodemographic factors were the independent variables analyzed. To achieve these objectives, the authors developed a questionnaire consisting of three sections for 18 years and above pilgrims. These sections are socio-demographic, visitor motivations, and satisfaction and loyalty to the visit. The socio-demographic part consists of 12 closed questions about the characteristics of pilgrims taken adapted from Lee et al. [58]. Section two is taken adopted from Pillai et al., [45] with a 5-point Likert scale ranging from 1 meaning not very important to 5 meaning very important. The Cronbach’s Alpha Coefficient of the motivation scale value reached 0.96, which means an acceptable internal consistency. Part three is about satisfaction and loyalty based on a 5-point Likert scale ranging from 1 means not very important and 5 means very important, and it is taken from Kim and Park [59].

Written informed consent was obtained from all participants in the questionnaires. This study is part of the Project ethically approved by the Research Dean of the ESPOL University. The questionnaire was prepared with Google Form. The authors conducted a pilot test of 25 surveys to validate the questions and find errors. After this pilot test, aspects of the wording of the questions were corrected so that they are better understood by the respondents.

The questionnaire was sent via WhatsApp in Bahrain, where most of the population are Muslims. The sample consisted of 384 valid surveys. The data collection period was from May to July 2021. A margin of error of +/− 5%, a confidence level of 95% and a variation of 50% were proposed. Then, data were organized, tabulated, and analyzed using SPSS Version 26 program. A factor analysis was applied to explain the relationship between the variables. The KMO index (Kaiser–Meyer–Olkin) and Bartlett’s test of sphericity were applied to know the appropriateness of performing factor analysis. The following stage used multiple regression to find the relationship between sociodemographic variables and the study variables (motivations, satisfaction, and loyalty). Only significant variables (p<0.05) were considered to find the relationship between variables.

## 5. Results

### 5.1. Sociodemographic profile of the sample surveyed

The present study was carried out in Bahrain where the majority of its population are Muslims and make the pilgrimage to the city of Mecca. The sample in terms of its sociodemographic characteristics was made up of national tourists (13.8%) and foreigners (86.2%). Also, 76.3% of tourists were men and 23.7% women. About marital status, 71.1% were married, while 16.9% were single. Regarding age, the majority group of tourists was between 21 and 30 years old with 39.1%, followed by those between 41 and 50 years old with 29.2%. Regarding the educational level, university students constituted the largest group (60.7%), followed by tourists with completed secondary studies (29.4%). Regarding professional activity, private employees (44.3%) and state employees (29.7%) made up the majority of the occupational groups in the sample. Most of the tourists (48.2%) had returned to the city of Mecca for the second time, followed by those who had visited it more than four times (25.4%). The majority group wanted to travel with their families (76.8%), followed by those who wanted to travel with their friends (14.8%). Most of the tourists stayed in the destination for four days and three nights (28.1%), or three days and two nights (29.1%) Finally, 36.5% of tourists claimed to have spent between USD 60,01 and USD 90 per day, followed by those who spent between USD 30,01 and USD 60 per day, with 24%.

### 5.2. Motivations in religious tourism

Factor analysis has been carried out to reduce the items to a smaller number of factors that facilitate interpreting the results. The Varimax rotation method was used to order the factors with the high and low factorial loads. The factors found were represented by 82.92% of the total variance. Cronbach’s Alpha of factors was between 0.987 and 0.935. Factorial loads were between 0.507 and 0.939, so all factorial loads were above the critical value of 0.50 suggested by Hair et al. [60]. The KMO index was 0.90, indicating an excellent relationship between the variables. In addition, Barlett’s test of sphericity was significant (p<0.05), so the factor analysis model was adequate. The results are shown in Table 1.

**Table 1:** Motivations in religious tourism (factor analysis).

Variable	Factor1: Religious	Factor2: Social and cultural	Factor 3: Shopping
To seek peace	0.939		
To appreciate/experience the grandeur of the churches	0.936		
To attend the religious festival	0.928		
To relieve daily stress	0.916		
To see Mecca	0.912		
To pay respect to the Saint’s relics	0.907		
To relieve boredom	0.906		
To escape from routine life	0.903		
To seek spiritual confort	0.889		
To share the experience with other believers/pilgrims	0.873		
To encounter religious fulfilment	0.869		
To experience the mystery of religion	0.867		
To redeem me	0.843		
To experience the holy atmosphere	0.775		
To spend a holiday	0.634		
To satisfy my curiosity		0.902	
To experience a different culture		0.84	
To fulfil a life-long desire		0.587	
To appreciate and experience ancient architecture		0.523	
To accompany friends or family		0.507	
To purchase religious items			0.848
To purchase local items			0.838
Cronbach’s α	0.987	0.830	0.935
Explained variance (%)	68.989	10.157	3.776
Cumulative explained variance (%)	68.989	79.147	82.922

According to the table, the first dimension has been called "religious" and is related to religious motivations, including seeking peace, attending religious festivals, appreciating or experiencing the grandeur of the churches, and relieving daily stress. This dimension included 68.99% of the total variance, making it the most important concerning the other factors. The second dimension has been called "social and cultural" and was related to the motivations to satisfy my curiosity, fulfill a lifelong desire, experience a different culture, appreciate and experience ancient architecture, and accompany friends or family. This dimension corresponded to 10.16% of the total variance. In contrast, the third dimension is "shopping", and it was related to the motivations to buy religious items and local items. This dimension included 3.78% of the total variance.

### 5.3. Relationship between sociodemographic variables and religious and social, and cultural motivations

To analyze the most important predictors in the "religious" and "social and cultural" motivations, the authors used the Multiple Enter Regression technique. Table 2 presents the results.

**Table 2:** Socio-demographic variables and religious, social, and cultural motivations.

	M1. Religious			M2. Social and cultural		
Variable	Beta	t	Sig.	Beta	T	Sig.
Age	0.077	1.395	0.164	-0.158	-3.067	0.002
Marital Status	-0.017	-0.325	0.745	-0.301	-6.312	0.000
Educational level	0.025	0.439	0.661	0.080	1.534	0.126
What was your average daily expenditure per person during this visit?	-0.066	-1.266	0.206	-0.153	-3.179	0.002
(Constant)		-0.380	0.704		3.027	0.003
F-statistic	0.973			16.395		
Sig.	0.000			0.000		
Tolerance (all items)	0.7 > T < 1			0.7 > T < 1		

In addition, there was no collinearity, with tolerance values between 0.7 and 1. No relationship was found between sociodemographic variables and "religious" motivation, so no sociodemographic variation has influenced religiously motivated tourists. Moreover, a negative relationship was found between "age" and "social and cultural" motivation (Beta = -0.158; p<0.002), so younger tourists were the most socially and culturally motivated. Likewise, a negative relationship has been found between "marital status" and "social and cultural" motivation (Beta = —0.301; p<0.000), so married tourists were the most motivated by social and cultural aspects. In addition, a negative relationship has been found between the "average daily expenditure per person" and the "social and cultural" motivation (Beta = —0.153; p<0.002), so the tourists who spent the least were the most socially and culturally motivated.

### 5.4. Relationship between sociodemographic variables and shopping motivation

A Multiple Enter Regression has been used to analyze the most important predictors in "shopping" motivation. The results are presented in Table 3.

**Table 3:** Socio-demographic variables and shopping motivation.

Variable	Beta	t	Sig.	Tolerance
Age	-0.155	-2.811	0.005	0.848
Marital Status	-0.023	-0.453	0.651	0.992
Educational level	0.022	0.394	0.694	0.830
What was your average daily expenditure per person during this visit?	-0.033	-0.651	0.515	0.972
(Constant)		1.214	0.226	
F-statistic	2.670			
Sig.	0.000			

The regression model was significant in the F test (p < 0.05). There was no collinearity, presenting tolerance values between 0.7 and 1. A negative relationship has been found between age and motivation for shopping (Beta = -0.155; p<0.005), so younger tourists were the most motivated by shopping. These findings have answered the research question Q1: What is the relationship of socio-demographic aspects with motivation to visit Mecca?

### 5.5. Relationship between sociodemographic variables and satisfaction

A Multiple Enter Regression has been used to analyze the most important predictors of satisfaction. Table 4 presents the results.

**Table 4:** Socio-demographic variables and satisfaction.

Variable	Beta	t	Sig.	Tolerance
Age	0.061	1.110	0.268	0.848
Marital Status	-0.061	-1.202	0.230	0.992
Educational level	0.072	1.301	0.194	0.830
What was your average daily expenditure per person during this visit?	-0.131	-2.545	0.011	0.972
(Constant)		17.809	0.000	
F-statistic	2.503			
Sig.	0.000			

The F test (p < 0.05) of the regression model was significant. Tolerance values were between 0.7, and no collinearity was found. Average daily spending per person and satisfaction (Beta = -0.131; p<0.011) presented a negative relationship, so the tourists who spent the least amount of money were the most satisfied. These findings answered the research question Q2: What is the relationship between socio-demographic aspects of pilgrims and satisfaction?

### 5.6. Relationship between sociodemographic variables and return and recommendation intentions

A Multiple Regression was used to analyze the most important predictors in return and recommendation. The results are presented in the Table 5.

**Table 5:** Socio-demographic variables and return and recommendation intentions.

	V1. Return			V2. Recommendation		
Variable	Beta	t	Sig.	Beta	t	Sig.
Age	0.057	1.028	0.305	0.061	1.112	0.267
Marital Status	-0.048	-0.943	0.346	-0.044	-0.858	0.391
Educational level	0.080	1.432	0.153	0.093	1.677	0.094
What was your average daily expenditure per person during this visit?	-0.126	-2.455	0.015	-0.136	-2.645	0.009
(Constant)		24.114	0.000		24.293	0.000
F-statistic	2.240			2.578		
Sig.	0.000			0.000		
Tolerance (all items)	0.7 > T < 1			0.7 > T < 1		

According to the results, the regression model was significant in the F test (p < 0.05), and there was no collinearity, presenting tolerance values between 0.7 and 1. A negative relationship was found between average daily expenditure per person and return intentions (Beta = -0.126; p<0.015). Likewise, a negative relationship was found between the average daily expenditure per person and the intentions of recommendation (Beta = -0.136; p<0.009), so that the tourists who spent the least were the ones who had the most intentions to return and recommend the destination.

### 5.7. Relationship between sociodemographic variables and saying positive things about destiny

To analyze the most important predictors in saying positive things about destiny, a Multiple Enter Regression has been used. The results are presented in the Table 6.

**Table 6:** Socio-demographic variables and saying positive about destiny.

Variable	Beta	t	Sig.	Tolerance
Age	0.069	1.264	0.207	0.848
Marital Status	-0.043	-0.840	0.402	0.992
Educational level	0.098	1.759	0.079	0.830
What was your average daily expenditure per person during this visit?	-0.156	-3.042	0.003	0.972
(Constant)		25.014	0.000	
F-statistic	3.222			
Sig.	0.013			

F test (p < 0.05) of the regression model was significant. Tolerance values between 0.7 and 1 and no collinearity was found. The average daily expenditure per person and saying positive things about the destination (Beta = -0.156; p<0.003) revealed a negative relationship so the tourists who spent the least were the ones who said the most positive things about the destination. These findings answered the research question Q3: What is the relationship between socio-demographic aspects of pilgrims and loyalty?

## 6. Discussion

The first objective of this study was to establish the motivational dimensions of the pilgrimage to Mecca. The research revealed three motivational factors: religious, social and cultural, and shopping. This finding is in line with previous studies about religious tourism. Piramanayagam et al., [37] identified the following motivational factors for visiting the Buddhist site of Bodhgaya: religious belief, architecture, culture, history, and service quality. Liro et al., [32] found that the visitors’ motives of the pilgrimage center in Krakow (Poland) are pilgrimage, prayers, enjoying a new place, participating in a cultural event, shopping, and business meeting. The contribution of this manuscript to the academic literature is by identifying three motivational factors for travelling to the holy city of Mecca.

Moreover, this study pointed out that religious motives are the most relevant factors for a visit, followed by non-religious motives such as social and cultural motives and shopping. For example, Božic et al. [61] study of Vujan Monastery (Serbia) found that visitors’ motives are divided into two types: Religious (seek for forgiveness, show and express love towards God, pilgrimage, pray at the tomb of saint and healing) and secular (cultural value, see the architecture, historical value and enjoy the nature surrounding the monastery). Similarly, Rybina [38] found that pilgrims from Kazakhstan, Kyrgyzstan, Tajikistan, and Uzbekistan visit Mecca for both religious (show love towards God, seek forgiveness and grow spiritually) and secular motives (learn about the culture and history of the sacred site).

This study aimed to identify the relationship between pilgrims’ socio-demographics aspects of and motivations as a second objective. Results showed that religious motivation (such as seeking peace, attending the religious festival, experiencing the holy atmosphere, and having the chance to see Mecca) does not depend on socio-demographic variables. Regarding social and cultural motivation, a negative relationship was found between age and those motivations. Younger visitors were most socially and culturally motivated. A negative relationship was also found between marital status and social and cultural motives, so married tourists were the most motivated. Moreover, a negative relationship was found between the average daily expenditure per person and the social and cultural motivation, so those visitors who spent the least were the most motivated socially and culturally. Finally, a negative relationship was found between age and shopping motivation regarding shopping motive. Therefore, younger tourists were the most motivated by shopping. These findings differ from Vistad et al. [44] about motives of pilgrimage to Nidaros, Norway, who found that solitude and meeting the locals/local heritage were more important for men than women. Education had no impact on motivational dimensions. Region/homeland influenced some motivational dimensions such as nature (knowledge and joy) being more important to other European pilgrims than to Norwegians while being in solitude was important to other European and German pilgrims than Norwegians. In another study by Irimias et al. [12] about the motives of Hungarians pilgrims to travel to religious sites in general and their relationship with their socio-demographic characteristics, there was a difference among age groups. For example, senior visitors travel to places where they can see religious sites, which is less important for young ones. Regarding faith, the motive of visiting for learning was more important to religious travelers than non-religious. Similarly, Pillai et al. [45] studied the impact of socio-demographic aspects on motivations to travel to St. Francis Xavier’s Holy Relics in Goa. They pointed out that socio-demographic aspects (age, gender, income, marital status, and education) did not influence on motivations (Experience religion, social Exploration, escape, experience belief, and shopping) to travel except for age. They found that those aged between 40 to 49 indicated that escape from daily routine was the most important motivation for travelling to sacred destinations. The discussion showed no inclusiveness in the results about the influence of socio-demographic aspects on motivations to travel to sacred sites. Therefore, there was an instant need to clarify this relationship in different religious destinations. Another contribution of this manuscript to the academic literature was finding negative relationship between some motives and socio-demographic aspects of visitors especially age, marital status, and the average daily expenditure per person.

The last objective of this research was to identify the relationship between socio-demographic aspects and variables of satisfaction and loyalty. This study has found a significant negative relationship between average daily spending per person and satisfaction regarding socio-demographic variables and satisfaction. Those results differ from the previous studies. For instance, Ahmad et al. [52] studied the impact of demographic aspects on the customer satisfaction of Malaysia Hajj Pilgrims. They found that gender, age, occupation and academic background had no significant difference in customer satisfaction. In contrast, Tatarusanu, et al. [54] investigated participating in the Saint Parascheva pilgrimage organized annually by Metropolitan Cathedral in Iasi, Romania. They found that only age and faith significantly impacted on satisfaction among the socio-demographic aspects of the study (age, education, area, income, gender, and faith). Regarding age, pilgrims aged 61 and above had the highest level of satisfaction, whereas the lowest level was among those younger than 20 years old. Concerning faith religious visitors who participated in the rituals had a high level of satisfaction compared to those who did not participate on regular bases and those who had no faith and did not participate.

In terms of loyalty, there is a paucity of studies conducted about the impact of socio-demographic aspects on it. This study has investigated three aspects: return intention, recommendation, and saying positive things. Generally, this study found a negative relationship between the average daily expenditure per person and loyalty. In detail, a negative relationship has been found between average daily expenditure per person and return intention, recommendation intentions, and saying positive things about the destination. In other words, visitors who spent the least were the ones who tended to return to the same destination, recommend the destination, and say positive things about the destination. Similarly, Bhat and Ahmad [55] studied tourists Jammu city for pilgrimage. They found that gender moderated the effect of destination image on satisfaction and loyalty of pilgrims. Thus, further studies should be conducted to understand the socio-demographic aspects impact on loyalty and generalize the results. Another contribution of this manuscript to the academic literature is the absence of negative relationship between socio-demographic aspects and satisfaction and loyalty variables. This relationship was found in the average daily expenditure per person variable.

In conclusion, more studies that relate socio-demographic variables and motivations are needed because the results of the available studies did not lead to conclusive results. The available studies and this one found that age and average daily expenditure per person have significant impact on motivations to travel to religious places. Likewise, average daily expenditure per person positively impacted the satisfaction and loyalty of these tourists.

Furthermore, the study has practical implications. First, the behavior disparities between older and younger religious tourists should be considered by all parties involved in managing religious sites. Therefore, it will be beneficial for service providers to segment those visitors according to their socio-demographic characteristics to ensure better services which can affect their satisfaction and loyalty. Hence, tourism should make improvements focused on tourists motivated by religion and on young tourists with offers related to social and cultural and shopping. Likewise, it should focus on tourists who want to spend less, with offers related to the social and cultural aspects since these tourists are the most satisfied and have greater loyalty to the destination. Moreover, this study can help managers of those religious sites in planning and implementing strategies that promote the importance of the religious site and enhance its position as a visitor’s attraction destination. Managers, marketers, and government representatives should advertise Mecca as a cultural and shopping hub in addition to a spiritual location. This announcement can be accomplished by promoting cultural events and Mecca retail areas like Souq Okaz (an old market that used to be hosted). Souq Okaz was recently recovered, and it has an array of cultural events including literary readings, exhibits of arts and crafts, and presentations. Other festivals and events can be organized throughout the year since lots of visitors visit Mecca around the year.

## 7. Conclusions

Religious tourism is a growing sector of the tourism market because of the many social and cultural changes in the 21st century. The interest in studying religious tourism stems from the fact that people live these days in a fast stead life resulting from the many changes in societies and thus tend to seek for calmness and spiritual growth. Pilgrimage centers worldwide are considered necessary for religious, heritage and cultural tourism. Despite pilgrimage centers’ popularity and increasing global importance, there is still a lack of knowledge about their dimensionality and impact of socio-demographic factors on the visit to these centers.

Among the main results of this study, we found three motivational dimensions for visiting the holy city of Mecca: religious, social and cultural, and shopping. Regarding socio-demographic aspects and motivations, the study found no relationship between those aspects and religious motivations. Concerning social and cultural motivation, a negative relationship has been found between socio-demographic variables (age, marital status, and average daily expenditure per person) and social and cultural motivation. Thus, younger tourists, married tourists, and tourists who spent less money were the most motivated by social and cultural aspects. Similarly, a negative relationship has been found between age and motivation for shopping. Thus, younger tourists were the most motivated by shopping. Moreover, this study found a negative relationship between average daily spending per person and satisfaction, so the tourists who spent the least were the most satisfied. Regarding loyalty, the authors found a negative relationship between average daily expenditure per person and other variables such as return intentions, recommendation intentions, and saying positive things about the destination. Hence, the tourists who spent the least were the ones who had intentions to return, recommend and say positive things about the destination.

Theoretically, the results mark a significant development in the investigation of Muslim tourists. Past research has focused mainly on the general motivation of the tourists with little significance attached to the Muslim family tourists, representing extensive and growing segments in tourism businesses. This study provides evidence for the experience effect by showing how respondents’ ages, marital status, and average daily expenditure per person affect the motivation of Muslim family visitors. Most importantly, this study is the first one that investigated the motivations, socio-demographic aspects, satisfaction, and loyalty of visitors to the holy city of Mecca since studies about those variables related to Islamic holy sites are scarce. Therefore, this study opens new doors for similar studies in the Islamic world and facilitates the understanding and nature of those destinations and visitors. This study discovered that socio-demographic factors significantly impacted visitor motivations, satisfaction, and loyalty particularly age and average daily expenditure per person. Findings provide significant knowledge to the literature on consumer behavior, Islamic tourism, motivation, satisfaction, and loyalty. This study strongly contributes to the academic literature because it is the first one that focused on Muslim family tourists to the city of Mecca in terms of their motivations, socio-demographic aspects, satisfaction, and loyalty.

The results of this study offer some important managerial implications to the marketers and authorities of the holy city of Mecca. It recommends that officials increase the religious motivations of these travelers by periodically researching their needs and organizing services to suit their desired spiritual experience. The results of this study could give the municipal of the city of Mecca insights of what motivates pilgrims to visit the holy destination of the Grand Mosque and this could encourage government to set strategies to ensure a better hosting of travelers. Also, to improve the social and cultural part, travel agencies and tourist companies to Mecca should promote social and cultural motivation among travelers in an appropriate way by providing service packages that involve visits to cultural and social sites such as museums and cultural centers. Also, to increase motivations for shopping, holy cities such as Mecca could build more shopping malls, traditional markets and restaurants offering high-quality local and international products, especially religious ones such as the Holy Quran, the rosary and dates. It could increase travelers’ satisfaction, make them prone to repeat visits, and recommend the destination to others. Services and activities in the city of Mecca should be varied to meet the different needs of different age groups, marital status, salary etc. Additionally, to enhance the social and cultural component, travel agents and tourism businesses in Mecca should appropriately encourage social and cultural motivation among visitors by offering service packages that include trips to social and cultural venues such museums and cultural centers. Additionally, holy places like Mecca might construct more malls, traditional markets, and restaurants that sell high-quality regional and worldwide goods, particularly religious ones like the Holy Quran, the rosary, and dates, in order to improve the motivation for shopping. It might boost visitors’ contentment, encourage them to return, and encourage them to recommend the place to others. Mecca’s services and events should be diverse to fulfill the requirements of people of all ages, marital statuses, income levels, and other categories.

This study has some limitations that should be taken into consideration. This research focuses on the visit to the holy city of Mecca. Other Islamic cities such as Najaf, Karbala, Mashhad Cairo, and Damascus can be investigated. In the future, other Islamic cities and mosques can be studied and the motivations for visit among them may be compared. However, this study’s main emphasis was the socio-demographic factors, motivations, satisfaction, and loyalty variables were the of. It will be necessary for the future to study additional factors, such as Islamic hospitality, Islamic tourist cultural values, Islamic beliefs, Islamic entertainment, Islamic morals, cultural variations, and how Muslims view tourism to improve Islamic tourism concepts globally. Data collection took time during spring. Thus, the impact of seasonality on the visit to the holy City of Mecca should be studied. Finally, because each country has its economic level, education, and age circumstances, the study’s context (Bahrain) limits the results’ generalizability to other countries for future research. Consequently, there can consequently be no inference regarding the findings’ applicability outside these situations. Moreover, using a country besides Bahrain aids our understanding, of why Muslims travel under specific circumstances.

## Supporting information

S1 File Survey on the motivations to visit Mecca. (DOC)

S1 Data (SAV)
