# Supplementary material for: Sociodemographic relationships of motivations, satisfaction, and loyalty in religious tourism: A study of the pilgrimage to the city Mecca
Source: PLoS One. 2023 Mar 30;18(3):e0283720. doi: 10.1371/journal.pone.0283720 (PMC10062575; doi:10.1371/journal.pone.0283720)
Supplement: S1 File — (DOC) [file pone.0283720.s001.doc]

**SURVEY ON THE MOTIVATIONS TO VISIT MECCA**

The ESPOL University of Ecuador is carrying out a study on the sociodemographic aspects and the motivations of religious tourism in the Pilgrimage to the city of Mecca (Saudi Arabia). Your authorization is requested to participate in this research project whose objective is to study religious tourism to develop the destination. Your participation is completely voluntary, if you do not wish to participate, there will be no negative consequences. You can withdraw from the study at any time. The response is completely anonymous. There is no associated risk. If you have any questions, you can consult Wilmer Carvache-Franco whose e-mail is: [wcarvach@espol.edu.ec](mailto:wcarvach@espol.edu.ec)

I agree to participate ❑

I don´t agree to participate ❑

Have you visited Mecca in the past 2 years? 

Are you over 18? 

**PERSONAL/SOCIO-DEMOGRAPHIC QUESTIONS**

**1. - Nationality: 1 ❑** Saudi  **2 ❑**  Foreign

**2. - Origin: 1 ❑** North American **2 ❑** European **3 ❑** South American

**4 ❑** Asian **5 ❑** Rest of the world

**3. - Country of origin_________________________________**

**4. - Gender: 1 ❑** Male **2 ❑**  Female

**5.- Marital status: 1** ❑ Single **2** ❑ Married **3** ❑ Others

**6.- Age: 1** ❑ Less than 20 years **2** ❑ 21-30 **3** ❑ 31-40 **4** ❑ 41-50 **5** ❑ 51-60 **6** ❑ More than 61 years

**7.- Educational level: 1** ❑ Primary **2** ❑ Secondary **3** ❑ University **4** ❑ Postgraduate / Master / Ph.D.

**8.- What is your occupation?**

**1** ❑ Student **2** ❑ Researcher/ Scientist **3** ❑ Businessman **4** ❑ Private Employee

**5** ❑ Public Employee **6** ❑ Pensioner **7** ❑ Unemployed **8** ❑ Other

**9.- How many times have you visited in Mecca?**

**1 ❑** First time **2 ❑ 2** times  **3 ❑ 3** times **4 ❑** More than 3 times

**TRAVEL QUESTIONS**

**10.- Who do you travel with?**

**1 ❑** Alone  **2 ❑** With your family **3 ❑** with friends **4 ❑** With your partner

**5 ❑** Others………

**11.- How many days did you stay in Mecca?**

**1** ❑ 1 day **2** ❑ 2 days and 1 night **3** ❑ 3 days and 2 night **4** ❑ 4 days and 3 nights

**5** ❑ 5 days and 4 nights **6** ❑ More than 5 days

**12.- What is your income level or monthly income (Dollars / month)?**

**1**  ❑ Less than $500 **2** ❑ From $501 to $1,000 **3** ❑ From $1,001 to $1,500

**4** ❑ From 1,501 to $2,000 **5** ❑ From $2,001 to $2,500 **6** ❑ From 2,501 to $3000 **7** ❑ More than $3000

**13.- What was your average daily expenditure per person in this visit? Including accommodation.**

**1**❑ Less than $30 **2**❑ $30,01-$60 **3**❑ $60,01 - $90 **4**❑ $90,01 - $120

**5**❑ $120,01- $150 **6**❑ More than $150

**MOTIVATION**

**14.- Rate from 1 to 5 (1 being little and 5 a lot) the reasons to visit** **this city. (Mark with an X)**

| **REASONS FOR VISITING MECCA** | **1** | **2** | **3** | **4** | **5** |
| --- | --- | --- | --- | --- | --- |
| To seek peace |  |  |  |  |  |
| To appreciate/experience the grandeur of the Kaaba |  |  |  |  |  |
| To seek spiritual comfort |  |  |  |  |  |
| To appreciate & experience ancient architecture |  |  |  |  |  |
| To experience the mystery of religion |  |  |  |  |  |
| To experience a different culture |  |  |  |  |  |
| To attend the Religious festival |  |  |  |  |  |
| Sightseeing |  |  |  |  |  |
| To share experience with other believers /pilgrim |  |  |  |  |  |
| To satisfy my curiosity |  |  |  |  |  |
| It’s a chance to see Mecca |  |  |  |  |  |
| For a holiday |  |  |  |  |  |
| To accompany friends or family |  |  |  |  |  |
| To escape from routine life |  |  |  |  |  |
| To relieve daily stress |  |  |  |  |  |
| To relieve boredom |  |  |  |  |  |
| For Religious fulfilment |  |  |  |  |  |
| To experience the holy atmosphere |  |  |  |  |  |
| To fulfil a life-long desire |  |  |  |  |  |
| To pay respect to the Umrah and Hajj relics |  |  |  |  |  |
| To redeem myself |  |  |  |  |  |
| To Purchase religious items |  |  |  |  |  |
| To Purchase local products |  |  |  |  |  |

**15. - Please rate from 1 to 5 (1 being little and 5 a lot) your overall satisfaction of this city. (Mark with an X)**

| **OVERALL VARIABLES** | **1** | **2** | **3** | **4** | **5** |
| --- | --- | --- | --- | --- | --- |
| 1. Overall satisfaction |  |  |  |  |  |
| 2. Satisfaction considering the invested time and effort |  |  |  |  |  |
| 3. Satisfaction in comparison with expectations |  |  |  |  |  |

**16. - Rate from 1 to 5 (1 being little and 5 a lot) your intention to visit again and recommend this city. (Mark with an X)**

| **RETURN AND RECOMMENDATION VARIABLES** | **1** | **2** | **3** | **4** | **5** |
| --- | --- | --- | --- | --- | --- |
| 1. I have the intention to revisit this city |  |  |  |  |  |
| 2. I have the intention to recommend this city |  |  |  |  |  |
| 3. When I talk about this city, I will give positive comments |  |  |  |  |  |
